# Combined Effects of Lactic Acid Bacteria Fermentation and Physical Milling on Physicochemical Properties of Glutinous Rice Flour and Texture of Glutinous Dumplings

**DOI:** 10.3390/foods14223882

**Published:** 2025-11-13

**Authors:** Jingyi Zhang, Bin Hong, Shan Zhang, Di Yuan, Shan Shan, Qi Wu, Shuwen Lu, Chuanying Ren

**Affiliations:** 1 Food Processing Research Institute, Heilongjiang Academy of Agricultural Sciences, Harbin 150086, China; 18846080235@139.com (J.Z.); gru.hb@163.com (B.H.); zhangshanfood@163.com (S.Z.); yuandi199707@163.com (D.Y.); 18845896856@163.com (S.S.); wuqi0322@163.com (Q.W.); 2 Heilongjiang Province Key Laboratory of Food Processing, Harbin 150086, China; 3 Heilongjiang Province Engineering Research Center of Whole Grain Nutritious Food, Harbin 150086, China

**Keywords:** milling methods, lactic acid bacteria fermentation, physicochemical properties, glutinous rice flour, glutinous dumplings

## Abstract

This study investigated the combined effects of lactic acid bacteria (LAB) fermentation and different milling methods (wet, semi-dry, and dry) on the physicochemical properties of glutinous rice flour (GRF) and the texture of the final product. A systematic analysis of rice samples treated with three LAB strains (*Lactiplantibacillus plantarum* CGMCC 1.12974, *Limosilactobacillus fermentum* CICC 22704, and *Lactobacillus acidophilus* CICC 22162) revealed that fermentation pretreatment created favorable conditions for subsequent physical milling by degrading the protein network and modifying the starch structure. The results demonstrated that fermentation combined with dry or semi-dry milling significantly improved the whiteness of GRF and the contents of γ-aminobutyric acid (GABA), total phenols, and total flavonoids, while reducing the contents of damaged starch (except in samples fermented with *Lb. acidophilus*) and protein by 2.91–12.43% and 17.80–32.09%, respectively. The functional properties of the GRF were also optimized: fermented flour exhibited higher peak viscosity, lower gelatinization temperature, and higher gelatinization enthalpy. Texture profile analysis revealed that glutinous dumplings prepared from fermented dry/semi-dry milled GRF, particularly those fermented with *Lp. plantarum*, showed significantly reduced hardness and chewiness, along with significantly improved cohesiveness and resilience. Consequently, their texture approximated that of high-standard wet-milled products. Correlation analysis based on the top ten discriminative features selected by random forest identified peak viscosity and breakdown viscosity as the most important positive factors associated with superior texture (high resilience, high cohesiveness, and low hardness), whereas damaged starch content and protein content were key negative correlates. In summary, this study confirms that the combination of fermentation and milling exerts a beneficial influence on the functional quality of GRF.

## 1. Introduction

As a characteristic traditional crop, glutinous rice holds a significant position in Asia, which is globally recognized for its high stickiness, soft texture, and excellent freeze–thaw stability [1]. These unique characteristics primarily stem from its amylopectin-dominant starch structure, which accounts for over 98% of the total starch content and is responsible for producing a delicate, soft gel texture upon thermal treatment [2]. For thousands of years, glutinous rice has served not only as a vital staple food but also as a key element embodying the essence of Asian dietary culture. Prior to being incorporated into food products, glutinous rice grains are dehulled and milled into flour. This flour is widely used in the preparation of both traditional and novel food items, including glutinous rice dumplings (tangyuan), rice cakes, mochi, and puddings. Glutinous rice flour (GRF) is conventionally produced through three milling routes: wet, dry, and semi-dry milling. Results indicate that the milling method significantly influences the compositional content and physicochemical properties of the resulting flour [3]. Wet milling, a traditional method for GRF production, involves soaking rice in water, draining, and then grinding with additional water. This process yields starch with higher purity and a lower degree of damage. However, it consumes substantial amounts of water and energy, and the washing step inevitably leads to significant loss of water-soluble nutrients, making it less environmentally friendly and more costly [4]. Dry milling involves grinding grains into flour under dry conditions without water addition. While simple and cost-effective, the high-speed grinding process generates a large amount of damaged starch, resulting in defects in the final product, such as excessive stickiness, hardened texture, and increased susceptibility to retrogradation [5]. The semi-dry milling method is characterized by the soaking of grains, draining of excess water, and final grinding. This method partially mitigates the issue of high damaged starch content but still struggles to completely avoid mechanical damage to starch granules [6].

There is consensus in the literature that the palatability of glutinous rice flour (GRF)-based foods is affected by the milling method. Consequently, pretreatment of glutinous rice prior to milling positively affects the properties of GRF and its products. Wang et al. [7] attributed the changes in GRF properties upon germination to structural modifications, including the disruption of its crystalline and short-range order, leading to increased swelling power but reduced batter viscosity and gel strength. Additionally, dumplings made from germinated flour exhibited improved texture. Employing an optimal heat-moisture treatment on GRF yielded taopian with superior textural profiles, as evidenced by its moderate hardness, ideal adhesiveness, and increased acceptability in the study by Xie et al. [8]. Another investigation by Qiu et al. [9] showed that pulsed electric field treatment applied to whole water-soaked glutinous rice grains resulted in flour with reduced gelatinization enthalpy and lower crystallinity. Furthermore, when ultrasonic treatment was implemented during the soaking process, Wang et al. [10] reported a progressive increase in the transparency of glutinous rice dumplings with increasing ultrasonic power, accompanied by gradual decreases in hardness, chewiness, and adhesiveness.

Among various modification methods, fermentation represents a well-established, cost-effective technique for food processing and shows significant potential in cereal modification. The fermentation process leverages microbial enzyme systems (e.g., proteases, amylases, and cellulases) to degrade cell walls and release nutrients, while simultaneously restructuring starch molecules via enzymatic and acidic activities [11]. This restructuring promotes cleavage of short-chain amylopectin branches, thereby increasing crystallinity and elevating the amylose-to-amylopectin ratio [12]. These modifications evidently alter the gelatinization, retrogradation, and textural properties of fermented rice, consequently influencing its application quality in various food products [13]. As a predominant component of natural microbiota and a critical probiotic starter, lactic acid bacteria (LAB) fermentation offers a compelling combination of safety, health benefits, and ability to impart unique flavors, contributing to its widespread application [14,15]. Studies on *Lactiplantibacillus plantarum*-fermented rice have shown that retrogradation of rice starch is inhibited, and the gelatinization temperature is reduced, making gelatinization easier [16]. Dou et al. [17] observed that LAB fermentation significantly improved the anti-staling properties of rice flour, thus improving the textural characteristics of fermented rice bread. Novotni et al. [18] found that rice flour sourdough prepared with *Limosilactobacillus fermentum* enhanced the specific volume and positively influenced the texture of bread.

Current research on GRF production has predominantly treated fermentation and milling as independent processes, leaving their interplay largely uninvestigated. We hypothesize that fermentation pretreatment alters the rice matrix structurally and compositionally, thereby creating favorable conditions for subsequent milling and enhancing GRF functionality. This study was designed to (i) systematically examine the influence of LAB fermentation and milling methods across multiple dimensions of GRF, including its physicochemical, nutritional, and functional properties; (ii) assess how these modifications determine the textural quality of end-products such as glutinous dumplings; and (iii) identify the key factors governing final product texture through multi-dimensional correlation analysis. By preparing GRF samples using three LAB strains (*Lactiplantibacillus plantarum* CGMCC 1.12974, *Limosilactobacillus fermentum* CICC 22704, and *Lactobacillus acidophilus* CICC 22162) in combination with varied milling techniques and characterizing their properties through a comprehensive analytical approach, this work clarifies the interactive effects of fermentation and milling, offering fundamental insights for the informed design of rice-based products.

## 2. Materials and Methods

### 2.1. Materials

*Lp. plantarum* CGMCC 1.12974 (China General Microbiological Culture Collection Center, Beijing, China, CGMCC), *Lb. acidophilus* CICC 22162, and *Lm. fermentum* CICC 22,704 (China Center of Industrial Culture Collection, Beijing, China, CICC) were used in this study. The raw material employed in this study was the glutinous rice cultivar Longjing 57, which was sourced from the Heilongjiang Academy of Agricultural Sciences (Harbin, China).

The frozen *Lp. plantarum*, *Lb. acidophilus*, and *Lm. fermentum* solutions were inoculated in MRS liquid medium using a 2% (*v*/*v*) inoculation and incubated at 37 °C, after which they were activated for three generations prior to experimental use. The *Lp. plantarum*, *Lb. acidophilus*, and *Lm. fermentum* suspensions were finally diluted to 10^8^–10^9^ cfu/mL in 0.85% NaCl solution.

A 100 g aliquot of glutinous rice was subjected to a 24 h soaking process in 150 mL of distilled water. For the fermented GRF, each strain was inoculated separately at a concentration of 2% (*v*/*m*) and fermented at 37 °C for 24 h, during which the pH dropped from an initial value of approximately 6.9 to a final value below 4.0. The naturally immersed samples were processed at room temperature as a control.

Subsequently, the rice was washed twice with distilled water and drained for milling in the different milling methods. After draining, the moisture contents of the respective groups were as follows: non-fermented = 34.57%, *Lp. plantarum* = 36.23%, *Lm. fermentum* = 36.01%, *Lb. acidophilus* = 35.48%. For the wet milling GRF, the rice grains with equal proportions of water were milled by a multi-use soup blender (max. speed: 42,000 rpm) (WFB-HS0454, Westinghouse, Pittsburgh, PA, USA) for 2 min. For the semi-dry milling GRF, the rice grains were milled using a universal pulverizer (max. speed: 20,000 rpm) (M 20 Universal mill, IKA, Staufen, Germany) for 2 min. For the dry milling GRF, the rice grains were equilibrated to ~10% moisture content by oven-drying (40 °C), and then milled using a universal pulverizer for 2 min. All rice flour was passed through a 50-mesh stainless-steel sieve for standardization and then dried to achieve a moisture content of approximately 10%.

### 2.2. Chemical Composition

Total starch, protein, reducing sugars, and total acidity were assayed following established procedures of enzymatic hydrolysis, Kjeldahl, DNS, and acid–base titration, respectively, while damaged starch was measured using a dedicated kit (K-SDAM, Megazyme, Bray, County Wicklow, Ireland) [19].

The sample extraction solution was prepared by 80% aqueous ethanol. A 1 g sample aliquot was combined with 10 mL of aqueous ethanol, subjected to 30 min ultrasonication, and subsequently centrifuged (3000× *g*, 5 min). The sample residue was extracted once with 10 mL of aqueous ethanol, and the solution extracted twice was mixed to a fixed volume of 25 mL. In accordance with Zeng et al. [20], total phenolics and flavonoids were quantified, with minor adaptations to the procedure. Determination of total phenols was performed with the Folin–Ciocalteu method. Specifically, 4 mL of sample or gallic acid standard was mixed with 1 mL Folin–Ciocalteu reagent and 4 mL of 12% sodium carbonate, then brought to 25 mL with 80% ethanol. After 60 min, the absorbance at 760 nm was recorded using a microplate reader (Synergy HTX, BioTek, Winooski, VT, USA). The aluminum nitrate colorimetric method was employed for total flavonoid content analysis. The assay procedure was as follows: 3 mL of sample or standard (rutin) was mixed with 300 µL of 5% NaNO_2_ (5 min incubation), then 300 µL of 10% Al(NO_3_)_3_ (6 min incubation), followed by 2 mL of 4% NaOH (12 min incubation). Recording of absorbance was performed on the Synergy HTX microplate reader at 510 nm. The calibration curve parameters for TPC and TFC assays are summarized in Table S1.

The determination of γ-aminobutyric acid (GABA) content was measured as previously described [21]. For sample analysis, the derivatization procedure was as follows: a 1.0 mL of the sample extraction solution or standard working solution was mixed with 0.20 mL of sodium bicarbonate solution (40 g/L) and 0.40 mL of 4-dimethylaminoazobenzene-4′-sulfonyl chloride solution (2 mg/L). The mixture was vortexed thoroughly and then incubated in a 70 °C water bath for 20 min to complete the derivatization reaction. Following cooling, the solution was filtered through a 0.22 µm membrane for HPLC analysis. GABA quantification was performed using an Ultimate 3000 HPLC system (Thermo Scientific, Waltham, MA, USA) equipped with a binary pump, degasser, and UV detector. Separation was achieved on a C18 column (Agilent Technologies, Santa Clara, CA, USA, 4.6 × 250 mm, 5 µm) maintained at 30 °C with a 1.0 mL/min flow rate, employing a mobile phase of acetonitrile: sodium acetate trihydrate (6.8 g/L) (35:65, *v*/*v*). Detection was conducted at 436 nm. The calibration curve parameters for the GABA assay are summarized in Table S1.

### 2.3. Properties of GRF

#### 2.3.1. Determination of Color of GRF

Color parameters (*L*, *a*, b*) of the GRF were quantified on a spectrophotometer (CM-5, Konica Minolta, Chiyoda-ku, Tokyo, Japan). The samples were placed under a plate glass for measurement. The Hunter whiteness was determined according to the method described by [22].

$$\mathrm{Hunter}\;\mathrm{whiteness}=100-\sqrt{a^{2}+b^{2}+{\left(100-L^{*}\right)}^{2}}$$

#### 2.3.2. Scanning Electron Microscopy (SEM)

The morphological features of different GRF samples were examined with a scanning electron microscope (Sigma 300, ZEISS, Oberkochen, Germany). Prior to SEM imaging, all GRF samples were affixed to aluminum stubs with conductive carbon tape and gold-coated to achieve proper conductivity. Micrographs were acquired at 3 kV and ×1000 magnification under high vacuum.

#### 2.3.3. Determination of Water Hydration Properties

We assessed three functional parameters: water absorption index (WAI), water solubility index (WSI), and swelling power (SP). Briefly, 0.1 g GRF (W_0_) was mixed with 20 mL of deionized water in a pre-weighed centrifuge tube (W_1_). The dispersion was subjected to two temperature regimes to assess both native and gelatinized starch behavior: it was agitated in a water bath at 25 °C for 30 min, and separately at 100 °C for 30 min with continuous shaking. Following centrifugation at 2000× *g* for 30 min, we collected the supernatant into a pre-weighed aluminum dish (W_2_) and dried it at 105 °C to constant weight (W_3_). The mass of the wet sediment with the tube was recorded as W_4_. The WAI, WSI, and SP values were derived based on the formulae in [23].

WAI = (W_4_ – W_1_)/W_0_

WSI (%) = (W_3_ – W_2_)/W_0_ × 100%

SP = (W_4_ – W_1_)/[W_0_ × (1 − WSI)]

#### 2.3.4. Rapid Visco Analysis

A Brookfield DV2T viscometer (Middleboro, MA, USA) was employed to characterize the pasting properties of GRF. The determination method followed the methodology of Wang et al. [24] with minor adjustments. 3 g of flour sample formed a suspension of 8% mass fraction. The testing profile included an initial hold at 50 °C (1 min), heating to 95 °C (5.5 °C/min), a hold at 95 °C (5 min), cooling to 50 °C (4.5 °C/min), and a final hold at 50 °C (3 min). The resulting pasting curve was analyzed to obtain key parameters, including peak viscosity (PV), trough viscosity (TV), final viscosity (FV), and pasting temperature (PT); alongside derived values defined as breakdown (BD = PV − TV) and setback (SB = FV − TV).

#### 2.3.5. Differential Scanning Calorimetry (DSC)

A differential scanning calorimeter (DSC Q2000, TA, New Castle, DE, USA) was employed to analyze the thermal behavior of GRF. A 6 mg sample mixed with deionized water at a 1:2 (*w*/*w*) ratio was loaded into a stainless-steel pan. The sealed pans were equilibrated at 4 °C for 24 h for complete hydration. Thermal analysis was carried out under nitrogen atmosphere (40 mL/min) to prevent condensation, following a heating program from 20 °C to 100 °C at 5 °C/min with an empty reference pan [25].

### 2.4. Preparation of Glutinous Dumpling

Dough was formulated with 90% water addition (based on flour weight), sealed in plastic film, and rested at 25 °C for 20 min. Subsequently, a 3.0 g portion of the dough was hand manipulated into a spherical shape, tightly wrapped with polyethylene film, and stored at −20 °C for 7 days in a freezer.

### 2.5. Evaluation of Glutinous Dumpling Quality

#### 2.5.1. Transparency of the Glutinous Dumpling Soups After Boiling

The optical transmittance of the dumpling cooking broth was determined at 620 nm. Five dumplings were boiled in 500 mL water for 6 min, the cooled broth was replenished to 500 mL, and measured on a UV-vis spectrophotometer (CARY 100, Varian, Palo Alto, CA, USA) with distilled water as a blank.

#### 2.5.2. Textural Properties of Cooked Glutinous Dumpling

The texture of cooked glutinous dumplings was analyzed at room temperature using a TA-XT Plus texture analyzer (Stable Micro Systems, Godalming, Surrey, UK) with a P/36R probe, following the methodology described by Wang et al. [26]. The texture analyzer was configured with the following speed settings: pretest 2.0 mm/s, test 1.0 mm/s, and posttest 1.0 mm/s. The deformation amount was set to 50%, and there were 5 s between two compressions. Using a trigger force of 5 g, the textural parameters of the dumplings were determined, comprising hardness, adhesiveness, springiness, cohesiveness, chewiness, and resilience.

### 2.6. Statistical Analysis

Triplicate measurements were conducted for all experiments, and the data were subsequently analyzed using GraphPad Prism 8 (GraphPad Software, San Diego, CA, USA). All statistical analyses were conducted using SPSS Statistics 19.0 (IBM, Chicago, IL, USA). After confirming significant differences by one-way ANOVA, post hoc pairwise comparisons were carried out using Waller–Duncan’s multiple range test. Statistical significance throughout this study was defined as *p* < 0.05. We identified the representative characteristics of different treatments using the randomForest package (v4.6-14) under default parameters. The clustered heatmap based on Pearson’s correlation coefficient was constructed and visualized through ComplexHeatmap package (v2.2.0).

## 3. Results and Discussion

### 3.1. Chemical Composition of Different GRF

As presented in Table S2, the milling methods significantly influenced the principal chemical constituents of GRF. Wet milling resulted in the highest total starch concentration of glutinous rice, followed by semi-dry and dry milling methods. Other component contents under wet milling were much lower than under other milling methods, which could be attributed to the loss of soluble compounds with water discharged during wet milling (*p* < 0.05) [27]. Wet milling had less damaged starch, and the content of damaged starch in W-N, S-N, and D-N was 3.15%, 4.09%, and 4.25%, respectively. This pattern can be explained by the pre-soaking step in wet and semi-dry milling, which swells and softens starch granules. As a result, they become more prone to breakage, whereas the intense mechanical and thermal forces in dry milling are well recognized to directly induce starch damage [5,23].

Under the same milling method, fermentation had no significant effect on the total starch content, but it had the effect of reducing the damaged starch content of wet milling (*p* < 0.05). The protein content decreases significantly after fermentation, which is attributed to the action of proteases produced by LAB (*p* < 0.05) [28,29,30]. These proteases break down proteins into small peptides or amino acids, which are utilized as a nitrogen source during bacterial growth, thereby reducing the protein content in fermented rice. The reducing sugar content in most GRF samples increased after fermentation, which can be attributed to the hydrolysis of starch into small-molecule sugars as the fermentation progressed. However, a decrease in reducing sugars was observed following fermentation with *Lp. plantarum*, likely due to its rapid consumption of the generated sugars for conversion into acids and other metabolites. This also accounts for the significant increase in its total acid content. After fermentation, the contents of total flavonoids, total phenols, and GABA in GRF were all increased, with more pronounced enhancements observed in samples fermented with *Lp. plantarum* and *Lm. fermentum* (*p* < 0.05). The enzyme system of LAB facilitates the release of bound phenolic and flavonoid compounds into their free forms [31]. These enzymes hydrolyze covalent bonds, thereby leading to an increase in both total phenolic and total flavonoid content. Previous studies have reported the phenomenon of increased GABA content by LAB, which is attributed to the fact that most LAB intrinsically possess glutamate decarboxylase (GAD). The enzyme is responsible for the decarboxylative reaction that transforms glutamate into GABA. However, due to variations in GAD enzyme activity among different bacterial strains, their capacity to produce GABA also differs significantly [32,33]. These findings are fully consistent with the established metabolic capabilities of LAB. As elaborated in contemporary literature, LAB fermentation can effectively enrich the bioactive profile of grain matrices through such diverse enzyme systems, providing a solid mechanistic basis for our observations [34].

### 3.2. Physicochemical Properties of Different GRFs

#### 3.2.1. Color Evaluation

The wet-milled GRF exhibited the highest Hunter whiteness value of 96.78, followed by the semi-dry milled and dry-milled samples with values of 92.72 and 92.29, respectively (Figure S1). This difference can be attributed to the greater mechanical and thermal energy input during the dry and semi-dry milling processes, which promotes the Maillard reaction, or browning [35]. Some studies have also suggested that the smaller average particle size of wet milled GRF provides a larger relative surface area, which reflects more light and consequently contributes to the observed increase in whiteness [36]. Compared to the non-fermented samples, the fermented samples exhibited an increase in whiteness, with improvements of 0.04–1.28% for dry-milled, 0.11–1.63% for semi-dry-milled, and 0.01–0.63% for wet-milled flour. The most pronounced enhancement was observed in samples fermented with *Lm. fermentum*. The observed effect resulted from the purification effect on starch achieved during fermentation, as evidenced by a significant reduction in protein content of 0.80–1.75 g/100 g after the fermentation process. Previous studies also found that with a decrease in protein content of GRF, the L* value increased significantly, while the b* value decreased significantly [37].

#### 3.2.2. Microstructure

**Figure 1:**
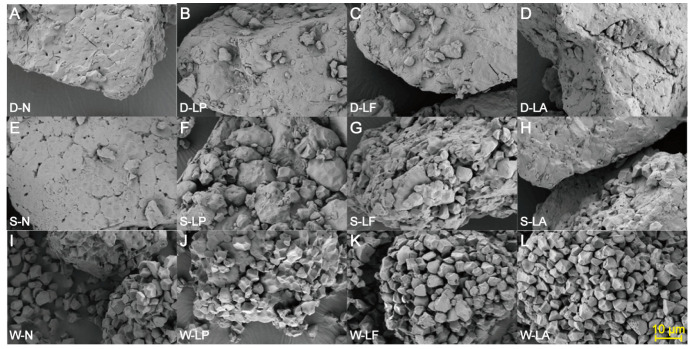
Morphology of dry milling (abbreviated as D) GRF (**A**–**D**), semi-dry milling (abbreviated as S) GRF (**E**–**H**), and wet milling (abbreviated as W) GRF (**I**–**L**) under different fermentation treatments. N: non-fermented; LP: *Lp. plantarum*; LF: *Lm. fermentum*; LA: *Lb. acidophilus*.

Dry-milled GRF particles exhibited irregular shapes and larger sizes with a rough surface, containing significantly larger agglomerated flour particles in which starch and protein were tightly bound (Figure 1). In contrast, semi-dry-milled samples showed partially exposed small particles. Compared to larger particles, wet-milled GRF demonstrated a markedly increased proportion of exposed small particles, presenting as small polygons with distinct edges and relatively smooth surfaces [38]. This observation aligns with previous characterization of glutinous rice starch granules—described as irregular polyhedrons with defined edges and an average particle size of approximately 6 μm [39]. The complete exposure of starch granules in wet-milled samples indicates substantial protein removal during the process, which can be attributed to the fact that water present in wet milling effectively weakens starch–protein interactions, thereby facilitating the release of intact starch granules from the endosperm [40].

Under the same milling method, while the overall morphology of flour particles remained largely unchanged after fermentation, the treatment increased the quantity of small particles in the GRF and induced pore formation on the surface of some granules. While such structural alterations have often been attributed to the action of organic acids produced by LAB [41], they may also result from the proteolytic activity of LAB-derived enzymes. In a related study on proso millet flour, Zhang et al. [42] noted that LAB fermentation caused surface etching, manifesting as pores and dents, which were ascribed to the hydrolysis of granule-associated proteins by microbial proteases. These surface defects likely facilitate the ingress of enzymes and promote the degradation of internal granular components [41]. These microstructural modifications directly contribute to the observed whitening effect: the formation of surface pores enhances light scattering, while the removal of protein layers unmasks the underlying starch granules, collectively increasing the overall light reflectance and thus the Hunter whiteness value of the fermented flour.

#### 3.2.3. Water Hydration Properties

As shown in Figure S2, the hydration properties revealed significantly lower WAI and SP values for W-N (3.37 and 3.01 g/g at 25 °C; 9.75 and 4.48 g/g at 100 °C) compared to other milling methods (Figure S2A,B,D,E). This phenomenon is principally accounted for by the reduced damaged starch content in wet-milled GRF. Mechanistically, damaged starch granules possess greater availability of hydroxyl groups for hydrogen bonding with water molecules [43,44,45].

WSI increased after fermentation, particularly under semi-dry milling conditions, with increases of 56.28–72.78% at 25 °C and 13.76–54.20% at 100 °C (Figure S2C,F). This enhancement can be attributed to the degradation of water-insoluble components, such as proteins and cell wall polysaccharides, by LAB-derived enzymes, which converts them into soluble substances [46]. Moreover, disintegration of the protein network facilitates the release of starch components into the aqueous phase, contributing to enhanced solubility [47]. Concurrently, both WAI and SP decreased after fermentation. The reduction in WAI aligns with the enzymatic breakdown of starch fragments and proteins, which diminishes their water-holding capacity [48]. The observed reduction in SP is likely due to the debranching of amylopectin induced by fermentation, which restrains its ability to absorb water and swell. This finding is consistent with the established understanding that a higher proportion of short-chain amylopectin, often resulting from debranching, is associated with a restrained SP [49]. Furthermore, the results of Hedayati and Niakousari [50] corroborate that the swelling capacity of starch granules is impaired by the disruption of chemical bonds.

#### 3.2.4. Pasting Properties

**Figure 2:**
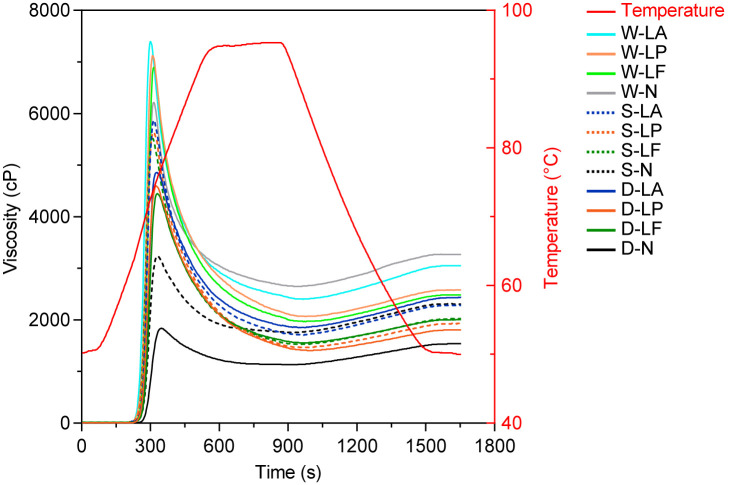
Changes in pasting properties of GRF under different treatments.

Figure 2 depicts the viscosity profile of GRF throughout thermal processing as determined by RVA. A comparison of different milling methods revealed that overall pasting viscosity parameters were highest in wet-milled GRF and lowest in dry-milled flour. This can be attributed to the lower heat generation during wet milling, as well as the smaller particle size, more intact granular structure, and reduced damaged starch content of wet-milled GRF, collectively resulting in superior viscosity properties compared to dry- and semi-dry-milled flours. Previous studies have also reported a link between viscosity and the degree of starch damage [19]. Furthermore, these properties are also influenced by the protein content in GRF. It is hypothesized that certain proteins in rice inhibit starch swelling during gelatinization; thus, higher protein content leads to lower viscosity [37]. These results align with the findings of Zhang et al. [3].

PV showed substantial enhancement following the fermentation process. Except for the wet-milled treatment, the TV and FV also increased after fermentation, which ameliorated the lower viscosity typically caused by dry and semi-dry milling. Among the treatments, *Lb. acidophilus* exhibited the most pronounced effect, followed by *Lp. plantarum* and *Lm. fermentum*. The observed viscosity enhancement is principally due to the ability of LAB metabolites to disrupt the protein–starch matrix and create structural defects that facilitate water penetration, as demonstrated by Wang et al. [51]. Consequently, the formation of hydrogen bonds with extended molecular chains is promoted, thereby enhancing viscosity. The pasting temperature decreased after fermentation, dropping from 75.9 °C, 74.4 °C, and 72.7 °C (unfermented) to 74.4–74.8 °C, 72.7–72.9 °C, and 72.2–72.5 °C, respectively, indicating that starch gelatinization occurred more readily. Fermentation lowers the starch pasting temperature due to enhanced hydration and an accelerated gelatinization process. This finding was corroborated by Dou et al. [17] who noted that partial protein decomposition reduces its inhibitory effect on starch gelatinization. SB quantifies the viscosity increment resulting from amylose realignment during cooling, reflecting the extent of starch retrogradation. A lower SB value signifies stronger anti-retrogradation properties. The fermented samples, especially those fermented with Lactobacillus plantarum, exhibited lower SB values (D-N = 400 cP, S-N = 466 cP, W-N = 516 cP) compared to the unfermented controls (D-LP = 409 cP, S-LP = 550 cP, W-LP = 617 cP), demonstrating superior anti-retrogradation characteristics.

#### 3.2.5. Thermodynamic Properties

**Figure 3:**
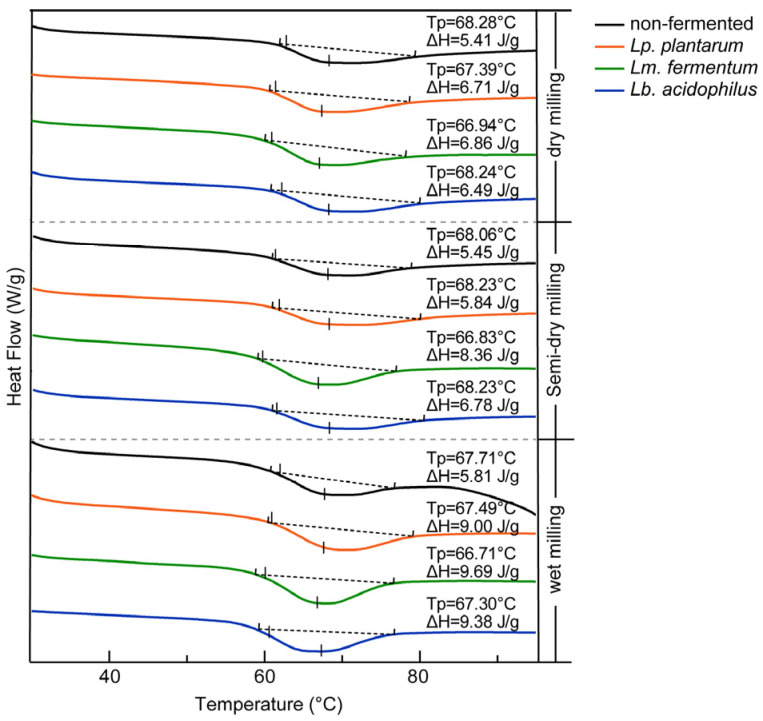
Changes in thermal properties of GRF under different treatments. The baseline used for peak integration is shown as a dashed line.

The gelatinization enthalpy (ΔH) represents the endothermic effect associated with the disintegration of ordered structures (crystalline regions) within starch granules during gelatinization, with its magnitude being directly correlated with starch crystallinity (Figure 3). The wet-milled flour exhibited the highest ΔH value (5.81 J/g), owing to its low damaged starch content and relatively intact granular structure [52]. Disrupting this well-organized structure demands greater energy input, resulting in a higher endothermic effect during gelatinization. In comparison, the semi-dry-milled flour (5.45 J/g) showed improved quality over the dry-milled flour (5.41 J/g), leading to a slightly higher ΔH value. Additionally, protein content also influences the enthalpy changes during starch gelatinization. Previous studies have similarly observed that a reduction in crude protein content contributes to an increase in ΔH [41].

In LAB-fermented GRF, the concurrent gelatinization temperature decrease and ΔH increase point to facilitated gelatinization and higher short-chain amylopectin proportion, corroborating RVA data. The universal ΔH enhancement in fermented samples reflects starch restructuring via amorphous domain degradation and molecular rearrangement. This finding is corroborated by Mao et al. [53]., whose research demonstrated that elevated short-chain amylopectin levels in fermented corn flour resulted in reduced pasting temperatures yet required greater gelatinization energy due to enhanced double-helical organization. These structural modifications resulted in increased crystallinity and a more ordered double-helical arrangement, thereby requiring greater energy input to disrupt the crystalline regions of the starch molecules [54].

### 3.3. Effects of Different GRF on the Cooking Quality of Prepared Glutinous Dumplings

#### 3.3.1. Transmittance of the Soup

**Figure 4:**
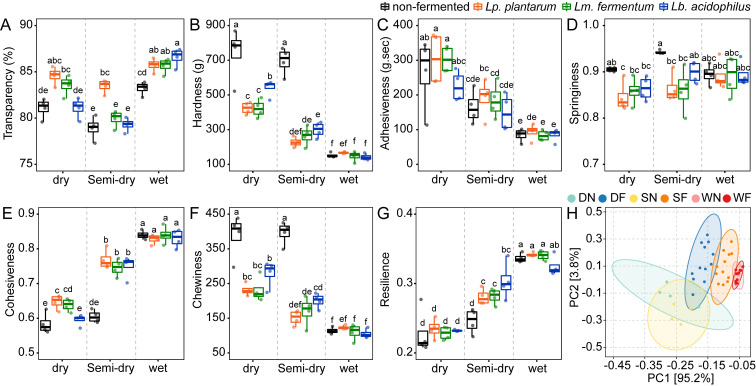
Changes in the quality attributes of GRF under different treatments (**A**–**G**) and the principal component analysis (PCA) of the samples based on these properties (**H**). D: Dry milling; S: Semi-dry milling; W: wet milling; N: non-fermented; F: fermented. Values with different superscript letters indicate significant differences at the level of *p* < 0.05.

The transmittance of cooked glutinous dumpling soup is an important quality indicator, as it reflects the degree of diffusion, dissolution, and dispersion of GRF in water during cooking. This parameter characterizes starch solubility while reflecting the dumpling’s capacity to preserve structural stability during cooking. As visualized in Figure 4A, the milling method had no significant effect on the soup transmittance, and W-N exhibited the highest light transmittance at 83.22%. However, fermentation with *Lp. plantarum* significantly increased the light transmittance within the same milling method, with an improvement of 3.00–5.73%, while *Lm. fermentum* and *Lb. acidophilus* also led to a slight increase. The elevated transmittance indicates that the fermented glutinous dumpling retained better morphological integrity during cooking, resulting in less leaching of solids into the soup and thus reducing turbidity.

#### 3.3.2. Textural Characteristics

Figure 4 also summarizes the textural properties of glutinous dumplings made from different GRFs. The high hardness (740.29 g) and chewiness (388.00) observed in dry-milled GRF (Figure 4B,F) can be explained by the substantial amount of damaged starch present. The damaged starch granules exhibit enhanced hydration capacity, thereby promoting the transformation of ordered segments in amylopectin molecules into tightly packed double helices and crystalline structures. This process accelerates the starch retrogradation process, resulting in enhanced gel rigidity [55]. In contrast, wet-milled GRF, due to its more intact starch granules, forms a soft and elastic gel with significantly higher cohesiveness (0.84) (Figure 4E), which is generally associated with slower retrogradation kinetics.

Fermentation treatment effectively improved the textural properties of dry- and semi-dry-milled samples, specifically manifested as significant reductions in hardness (26.89–67.32%) and chewiness (28.00–61.80%), along with an increase in cohesiveness (3.45–28.33%). The observed trend of textural improvement is consistent with reports in existing literature. According to these studies, the acidic conditions generated during fermentation can inhibit starch molecule realignment, thereby forming a softer gel with improved anti-retrogradation properties [56,57]. Additionally, the pore formation induced by fermentation, as observed in the microstructure, may also contribute to the textural softening, as starch granules with higher pore density tend to exhibit a softer texture [58]. These factors collectively provide a plausible explanation for this phenomenon. Principal component analysis results (Figure 4H) visually confirm this point, as the fermented samples shifted toward the wet-milled control group in terms of textural properties, indicating that fermentation precisely regulates the assembly of the gel network by modifying the starch matrix.

**Figure 5:**
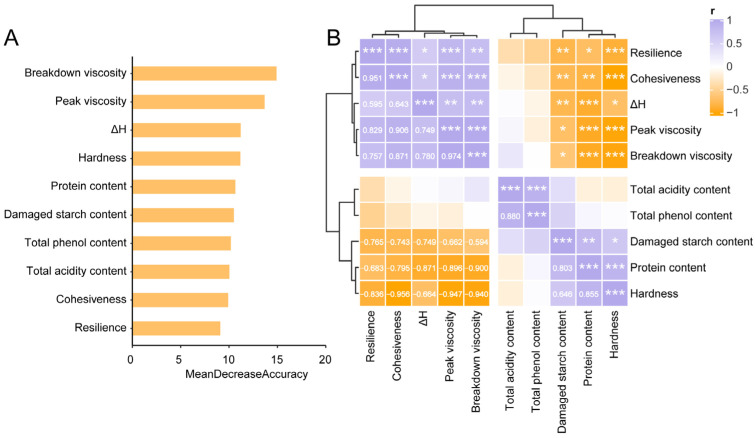
Analysis of the top 10 indicator features by random forest modeling (**A**) and their correlation heatmap (**B**). *p* values were calculated using Pearson’s rank correlation test, * *p* < 0.05; ** *p* < 0.01; *** *p* < 0.001.

As shown in Figure 5, we employed the random forest algorithm to identify the top ten most important distinctive features, and the correlations between these ten GRF properties and the quality of glutinous dumplings were analyzed. As illustrated in Figure 5B, BD and PV received the highest importance scores. They showed significant positive correlations with resilience (*r* = 0.757 and 0.829), cohesiveness (*r* = 0.871 and 0.906), and ΔH (*r* = 0.780 and 0.749), but were negatively correlated with damaged starch (*r* = −0.594 and −0.662) and protein content (*r* = −0.900 and -0.896) (*p* < 0.05). Hardness, as the primary textural indicator of glutinous dumplings, was significantly positively correlated with damaged starch and protein content. GRF with lower damaged starch and protein content exhibited higher pasting viscosity and ΔH, which may be more conducive to the texture of glutinous dumplings. Furthermore, fermentation significantly reduced protein content and slightly decreased damaged starch content. Fermentation with *Lp. plantarum* reduced protein and damaged starch content by 25.82–30.92% and 0.25–33.47%, respectively, while *Lm. fermentum* reduced them by 24.17–29.77% and 3.41–38.15%, respectively.

## 4. Conclusions

This study confirms the efficacy of LAB fermentation as a pretreatment method for enhancing dry- and semi-dry-milled GRF quality. The fermentation process degrades the glutinous rice protein network, creating favorable conditions for subsequent physical milling and significantly enhancing the overall flour quality: it increases the content of bioactive components in terms of nutritional composition, while reducing starch damage and improving pasting properties in terms of processing characteristics. Most importantly, the optimized flour produced textural properties in the final product comparable to high-cost wet-milled flour, with notable improvements in key parameters including hardness, cohesiveness, and resilience. These findings offer the traditional rice flour industry a new approach to balance cost control with quality enhancement, enabling producers to significantly improve product quality through bioprocessing technology without substantially increasing production costs. It is worth noting that, while lactic acid bacteria fermentation improves product quality, its metabolites may also broaden the product’s application potential in the field of food preservation. For example, the study by La Bella et al. [59] demonstrated that the symbiotic fermentation broth of lactic acid bacteria and cyanobacteria exhibits broad-spectrum and sustainable biocontrol potential, providing new insights for developing more sustainable food processing solutions in the future.

## Data Availability

The original contributions presented in this study are included in the article/Supplementary Materials. Further inquiries can be directed to the corresponding authors.

## Supplementary Materials

The following supporting information can be downloaded at: https://www.mdpi.com/article/10.3390/foods14223882/s1, Table S1: Calibration curve parameters for the quantification of total phenolics, flavonoids, and GABA; Table S2: Changes in chemical composition of GRF under different treatments; Figure S1: The hunter whiteness of GRF under different treatments; Figure S2: Changes in water hydration properties of GRF at 25°C (A–C) and 100°C (D–F) under different treatments.
