# Supplementary material for: Combined Effects of Lactic Acid Bacteria Fermentation and Physical Milling on Physicochemical Properties of Glutinous Rice Flour and Texture of Glutinous Dumplings
Source: Foods. 2025 Nov 13;14(22):3882. doi: 10.3390/foods14223882 (PMC12651270; doi:10.3390/foods14223882)
Supplement: Supplementary file 1 [file foods-14-03882-s001.zip › foods-3956500-supplementary.pdf]

Combined Effects of Lactic Acid Bacteria Fermentation and Physical Milling on Physicochemical Properties of Glutinous Rice Flour and Texture of Glutinous Dumplings

Jingyi Zhang [1,2,3], Bin Hong [1], Shan Zhang [1], Di Yuan [1], Shan Shan [1], Qi Wu [1], Shuwen Lu [1,*] and Chuanying Ren [1,*]

1 Food Processing Research Institute, Heilongjiang Academy of Agricultural Sciences, Harbin 150086, China;
18846080235@139.com (J.Z.); gru.hb@163.com (B.H.); zhangshanfood@163.com (S.Z.); yuandi199707@163.com (D.Y.); 18845896856@163.com (S.S.); wuqi0322@163.com (Q.W.)

2 Heilongjiang Province Key Laboratory of Food Processing, Harbin 150086, China

3 Heilongjiang Province Engineering Research Center of Whole Grain Nutritious Food, Harbin 150086, China

* Correspondence: shuwenl@sina.com (S.L.); chuanying1023@163.com (C.R.)

Supplementary Tables

Table S1. Calibration curve parameters for the quantification of total phenolics, flavonoids, and GABA.

Analyte	Method	Standard Used	Concentration Range	Regression Equation	R^2 Value
Total Phenolics	Spectrophotometric	Gallic Acid (Solarbio, SG8040, HPLC≥ 98%)	0 - 20 mg/mL	y = 2.4228x - 0.0054	0.9998
Total Flavonoids	Spectrophotometric	Rutin (Solarbio, SR8250, HPLC≥ 98%)	0 - 20 mg/mL	y = 1.2669x - 0.0074	0.9995
GABA	HPLC	GABA (Sigma-Aldrich, 03835, HPLC ≥97%)	0 - 50 μg/mL	y = 0.2875x + 0.2126	0.9833

Table S2. Changes in chemical composition of GRF under different treatments.

	total starch content (g/100 g)	damaged starch content (g/100 g)	protein content (g/100 g)	reducing sugar content (mg/g)	total phenol content (mg/g)	GABA content (mg/100 g)	total flavonoid content (mg/g)	total acidity content (mg lactic acid/g)
D-N	87.37±0.81^{c}	4.25±0.58^{b}	7.41±0.13^{a}	2.23±0.03^{f}	0.33±0.00^{g}	nd	0.15±0.01^{c}	0.37±0.04^{h}
D-LP	88.59±1.00^{bc}	4.13±0.05^{bc}	5.66±0.09^{d}	1.56±0.03^{h}	0.46±0.01^{a}	0.72±0.11^{a}	0.19±0.03^{a}	4.46±0.04^{a}
D-LF	88.28±0.94^{bc}	4.11±0.09^{bc}	5.71±0.07^{d}	2.43±0.09^{e}	0.41±0.00^{c}	0.62±0.11^{a}	0.18±0.02^{ab}	4.02±0.04^{c}
D-LA	87.54±0.76^{c}	4.69±0.48^{a}	6.17±0.05^{c}	6.29±0.03^{b}	0.36±0.01^{f}	0.05±0.08^{b}	0.14±0.01^{c}	2.99±0.04^{e}
S-N	87.55±1.04^{c}	4.09±0.13^{bc}	7.09±0.16^{b}	2.03±0.03^{g}	0.27±0.01^{j}	nd	0.10±0.01^{ef}	0.34±0.04^{h}
S-LP	88.74±1.19^{bc}	4.08±0.06^{bc}	5.61±0.03^{d}	3.35±0.03^{d}	0.38±0.01^{e}	0.85±0.20^{a}	0.13±0.01^{cd}	4.31±0.04^{b}
S-LF	87.73±0.82^{c}	3.78±0.06^{c}	5.71±0.22^{d}	4.02±0.06^{c}	0.42±0.01^{b}	0.64±0.23^{a}	0.16±0.01^{bc}	3.38±0.04^{d}
S-LA	87.67±0.91^{c}	4.90±0.06^{a}	6.29±0.02^{c}	10.50±0.04^{a}	0.39±0.00^{d}	0.08±0.18^{b}	0.14±0.00^{c}	3.01±0.04^{e}
W-N	89.45±0.88^{ab}	3.15±0.07^{d}	5.75±0.24^{d}	2.05±0.03^{g}	0.30±0.00^{i}	nd	0.07±0.00^{g}	0.32±0.00^{h}
W-LP	89.62±0.83^{ab}	2.36±0.08^{e}	4.57±0.04^{e}	1.52±0.06^{h}	0.31±0.01^{h}	nd	0.11±0.00^{ef}	1.84±0.04^{f}
W-LF	90.56±0.95^{a}	2.28±0.04^{e}	4.21±0.03^{f}	2.42±0.03^{e}	0.33±0.00^{g}	nd	0.11±0.01^{de}	1.79±0.00^{f}
W-LA	87.41±1.00^{c}	2.44±0.05^{e}	4.16±0.11^{f}	3.95±0.10^{c}	0.27±0.00^{j}	nd	0.08±0.00^{fg}	1.18±0.04^{g}

Mean value ± SD with different superscript letters in the same column are significantly different ($p < 0.05$).
nd, non-detected.

Supplementary Figures

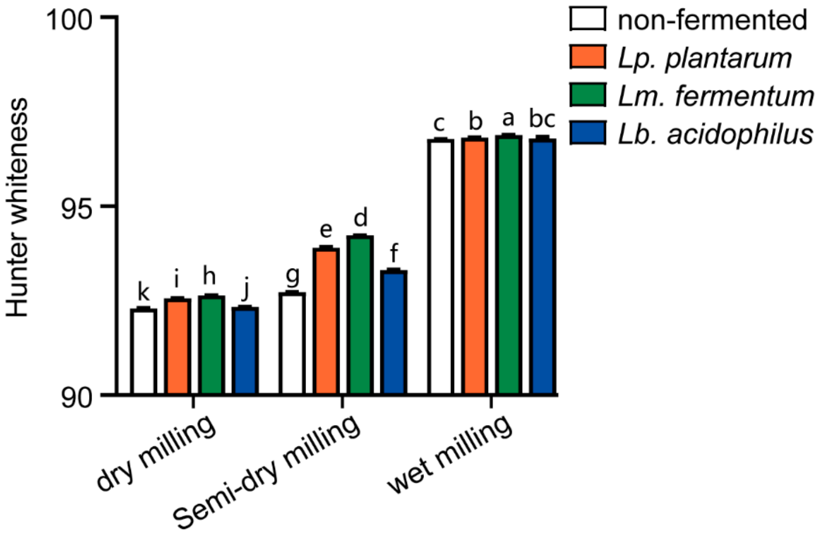

Figure S1. The hunter whiteness of GRF under different treatments. Values with different superscript letters indicate significant differences at the level of $p < 0.05$.

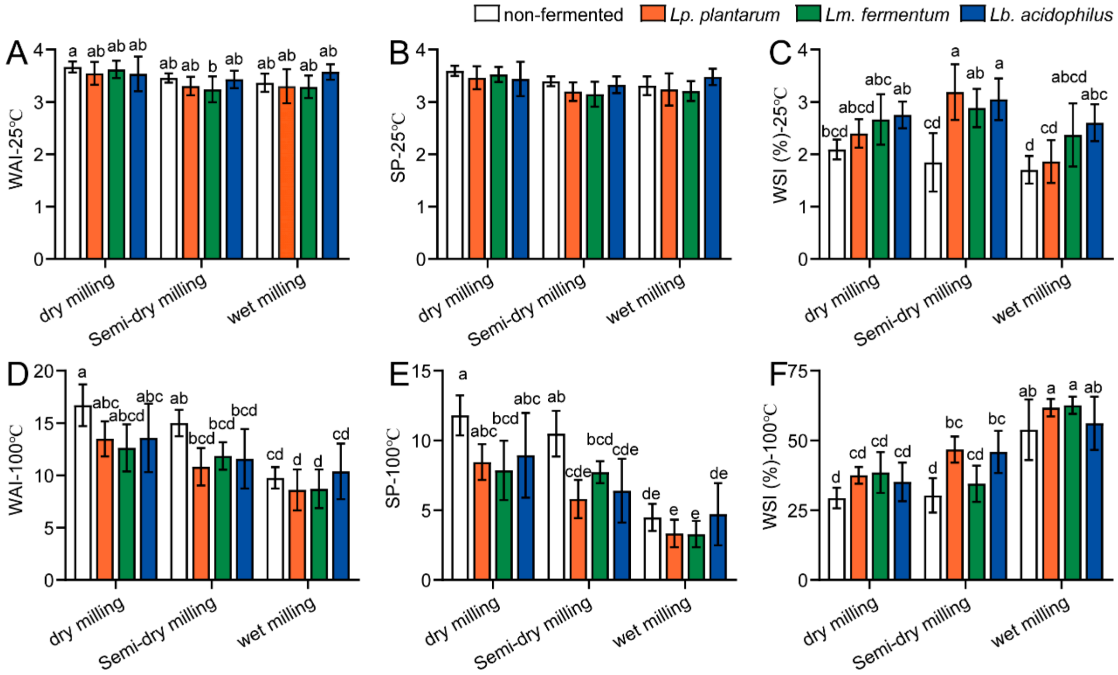

Figure S2. Changes in water hydration properties of GRF at 25°C (A–C) and 100°C (D–F) under different treatments. Values with different superscript letters indicate significant differences at the level of $p < 0.05$.
